# Tri-arabinosylation facilitates the bioactivity of CLE3 peptide in *Arabidopsis*

**DOI:** 10.5511/plantbiotechnology.25.0120b

**Published:** 2025-06-25

**Authors:** Satoru Nakagami, Taiki Kajiwara, Hajime Hibino, Taku Yoshiya, Masayoshi Mochizuki, Shugo Tsuda, Toshihiro Yamamoto, Shinichiro Sawa

**Affiliations:** 1 Graduate School of Science and Technology, Kumamoto University; 2 Peptide Institute, Inc.; 3 Graduate School of Science, Osaka University; 4 International Research Center for Agricultural and Environmental Biology, Kumamoto University; 5 International Research Organization for Advanced Science and Technology (IROAST), Kumamoto University; 6 Institute of Industrial Nanomaterials (IINa), Kumamoto University

**Keywords:** Arabidopsis, CLE peptide, post-translational modification, shoot apical meristem

## Abstract

Post-translational modification is critical for the bioactivity of small secreted-signaling peptides. The shoot apical meristem (SAM) activity that defines SAM size is controlled by the CLAVATA3 (CLV3) peptide ligand, which belongs to the CLV3/EMBRYO SURROUNDING REGIONRELATED (CLE) family, and its cognate receptor CLV1. The mature CLV3 peptide is post-translationally modified with tri-arabinose, increasing the binding affinity with CLV1. However, the mature form of most CLE peptides is unknown. Here we apply the synthetic CLE3 peptide with tri-arabinose to *clv3* mutant to determine whether the CLE3 peptide can reduce the SAM size. We show that tri-arabinosylated CLE3 peptide exhibits stronger bioactivity in the SAM in a CLV1/BAM1-dependent manner. Our data emphasizes the importance of post-translational modification on peptide signaling, helping to characterize bona fide mature peptides.

Small secreted-signaling peptides are essential for cell-to-cell communication to coordinate cellular functions in multicellular organisms. The CLAVATA3 (CLV3)/EMBRYO SURROUNDING REGIONRELATED (CLE) family is one of the largest signaling peptide families in plants. In a model plant *Arabidopsis thaliana*, 32 *CLE* genes are contained in the genome and encode 27 distinct CLE peptides (Jun et al. 2008). Accumulated evidence has shown that CLE peptides act in various aspects of physiological responses and plant-microbe interactions (Fletcher 2020; Nakagami et al. 2024). CLE prepropeptides are post-translationally processed and modified, resulting in a mature peptide. Secreted-mature peptides are recognized by plasma membrane-localized receptor complexes, thereby provoking an intracellular signaling cascade.

Canonical CLE prepropeptides possess an N-terminus signal peptide, a C-terminus conserved CLE domain, and a variable domain between the signal peptide and the CLE domain (Betsuyaku et al. 2011). CLE prepropeptides are cleaved by a signal peptide peptidase, resulting in a propeptide. Propeptides are subjected to post-translational modifications and proteolytic cleavages to generate a mature peptide in length of 12–14 amino acids. For instance, the mature CLV3 peptide has two distinct forms; a 12 amino acids peptide with hydroxyprolines (Hyp) in the 4th and 7th positions and a 13 amino acids peptide with Hyp at the 4th position and tri-arabinosylated Hyp at the 7th position, containing an additional histidine residue at the 13th position (Kondo et al. 2006; Ohyama et al. 2009). The 13 amino acids form of CLV3 without tri-arabinosylation can directly bind to the extracellular domain of CLV1 with a slight binding affinity, while the tri-arabinosylated one shows stronger binding affinity, indicating that the tri-arabinosylation is critical for the binding affinity between CLV3 peptide and CLV1 receptor (Ohyama et al. 2009). In the shoot apical meristem (SAM), the expression of the *WUSCHEL* (WUS) gene that promotes stem cell activity is repressed by the CLV3-receptor module, thereby maintaining SAM size. Among the 27 distinct CLE peptides, tri-arabinosylation has been discovered only from CLE2 and CLV3 peptides, which belong to groups 1A and 1B respectively (Ohyama et al. 2009; Zhang et al. 2020).

Genetic studies have shown that *CLE3* gene, which encodes the group 1A peptide, is involved in lateral root formation, root-knot nematode infection, sugar homeostasis, and immune response (Araya et al. 2014; Ma et al. 2020, 2022; Nakagami et al. 2023a, b; Okamoto et al. 2022). However, due to technical obstacles, whether the mature form of CLE3 peptide contains the tri-arabinose moieties remains uncharacterized. To examine the contribution of tri-arabinosylation to the bioactivity of CLE3 peptide, we utilized artificially synthesized CLE3 peptides with (ARA3-CLE3p) or without (CLE3p) tri-arabinosylation and the bioassay system using the mutant lacking *CLV3* that has enlarged SAM due to SAM homeostasis defect (Figure 1A). The ARA3-CLE3p was synthesized using a standard Fmoc-based solid-phase peptide synthesis method with Fmoc-Hyp(Ara)_3_ (Supplementary Figure S1) (Kaeothip et al. 2013). The synthetic peptides were applied to *clv3-8* seeds after stratification for 2 days. The SAM areas were measured at 7 days post-treatment (Figure 1B). Consistent with previous studies, the SAM area of *clv3-8* was significantly larger than that of the wild-type under mock treatment. The SAM of *clv3-8* treated with 100 nM CLE3p exhibited no significant difference compared to that of mock-treated *clv3-8*, whereas 1 µM or 10 µM CLE3p was able to reduce the SAM size with 10 µM CLE3p showing greater effect (Figure 2, Supplementary Figure S2). Consistent with this trend, the SAM size of *clv3-8* treated with ARA3-CLE3p was reduced in a dose-dependent manner (Figure 2, Supplementary Figure S2). The SAM area of ARA3-CLE3p-treated *clv3-8* was smaller than that of CLE3p-treated seedlings at the same dosage (Figure 2B), demonstrating that tri-arabinosylation facilitates the bioactivity of CLE3 peptide. A previous study has shown that tri-arabinosylated-CLV3 peptide does not affect the SAM size of the *clv1 bam1* double mutant which lacks the two receptor kinases (Shinohara and Matsubayashi 2015). We therefore tested whether ARA3-CLE3p reduces the SAM size in the *clv1-101 bam1-3* mutant. As expected, *clv1-101 bam1-3* treated with ARA3-CLE3p exhibited no significant differences in the SAM size compared to mock treatment (Figure 3). Similar trends have been observed in CLE2 and CLV3 peptides (Ohyama et al. 2009; Shinohara and Matsubayashi 2015), therefore, the tri-arabinose moiety within the CLE3 peptide may contribute to the binding affinity between the CLE3 peptide ligand and CLV1/BAM1 receptors.

**Figure 1:**
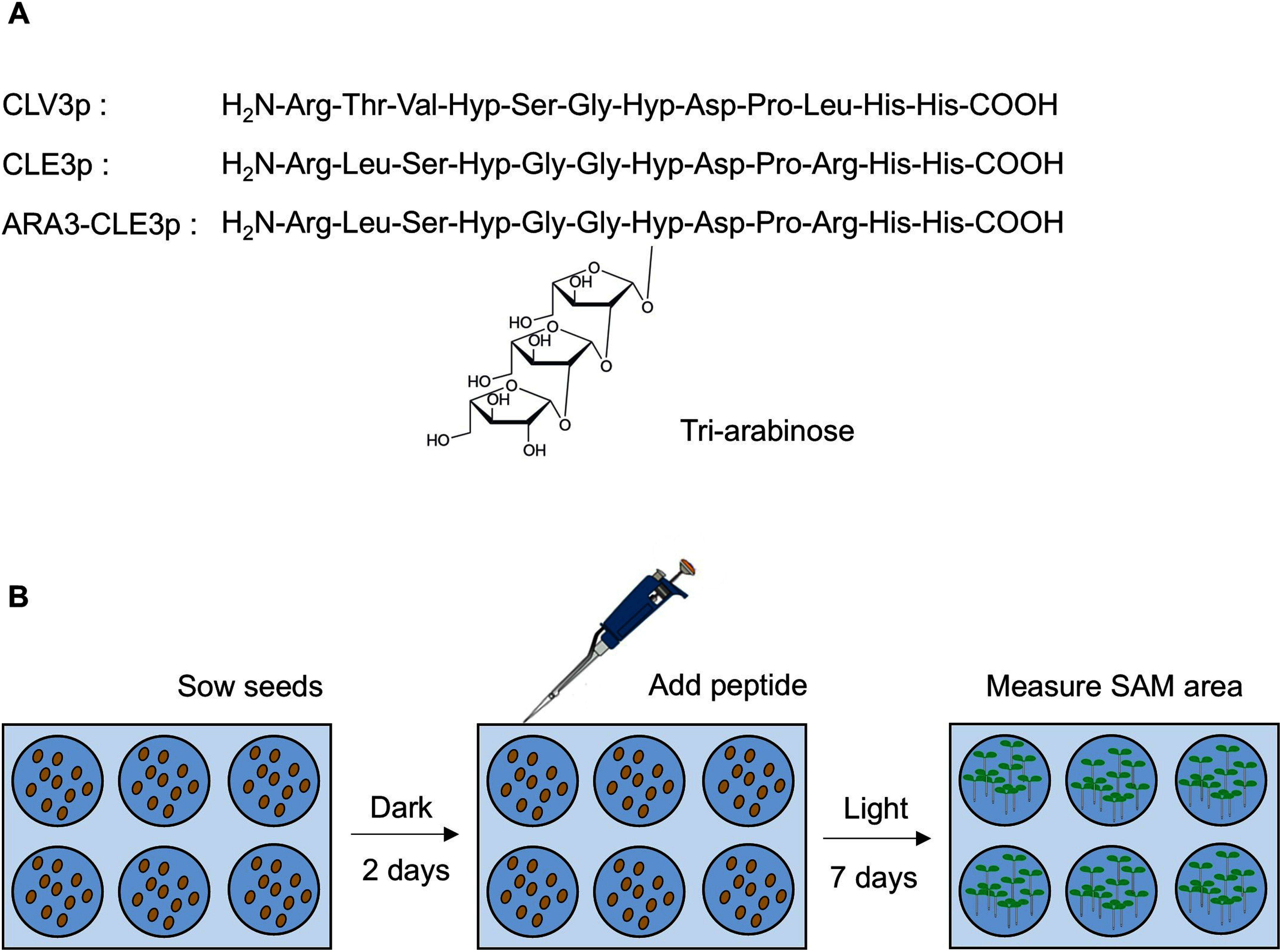
Figure 1. Experimental setting for testing synthetic peptides bioactivity. (A) Amino acid sequences of synthetic peptide ligands for the SAM reduction assay. Hyp: hydroxyproline. (B) Experimental design of SAM reduction assay for testing synthetic peptides bioactivity. Surface-sterilized seeds were stratified for 2 days. Synthetic peptides solved in ultrapure water were added to the wells and incubated under continuous light conditions at 23°C for 7 days. Then, the SAM areas were measured and quantified.

**Figure 2:**
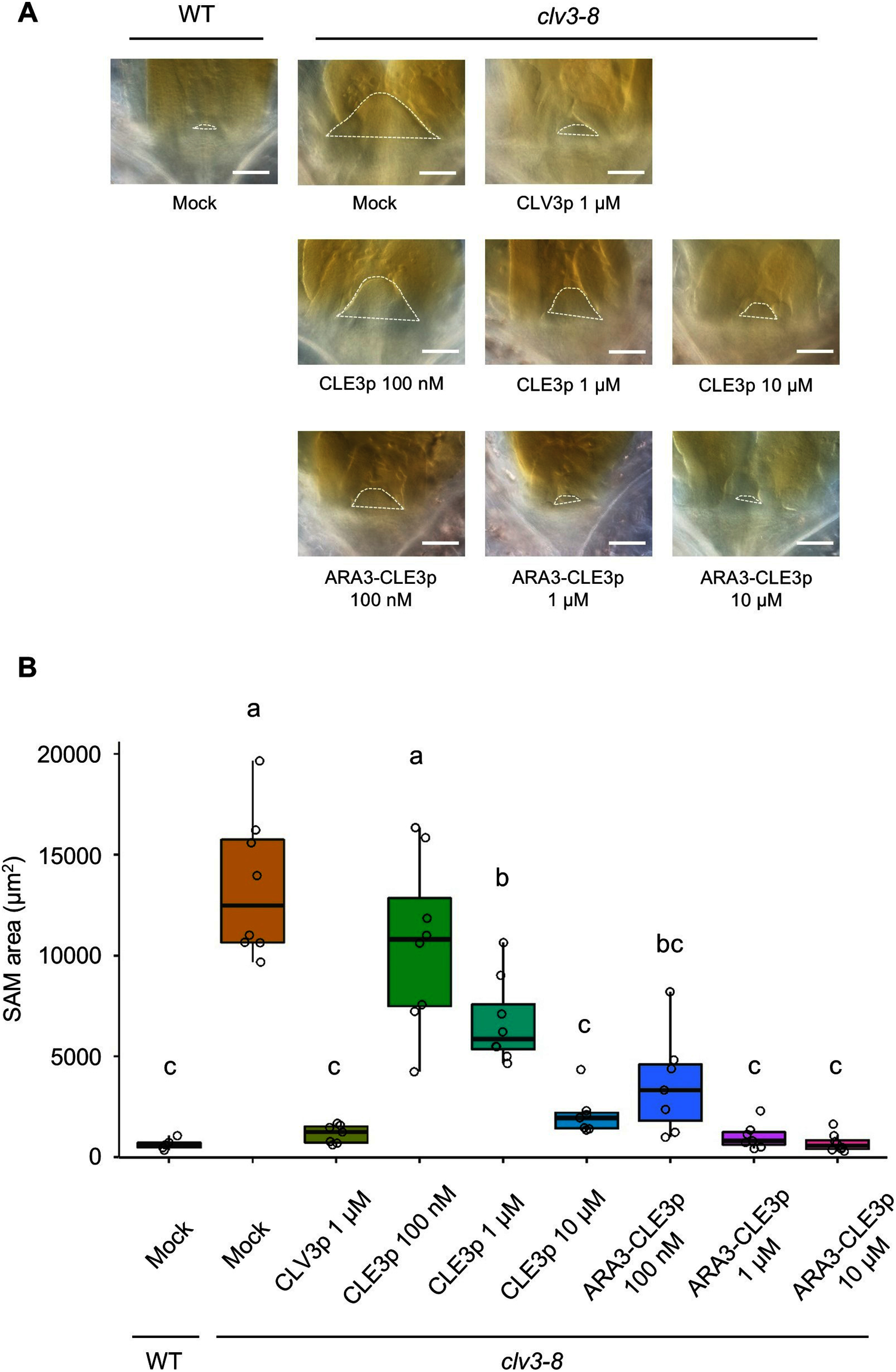
Figure 2. SAM reduction assay using synthetic peptides. (A) Representative micrographs of the SAMs of the wild-type (WT) and *clv3-8* seedlings treated with synthetic peptides. Broken lines denote SAM areas. Bar=100 µm. (B) Quantified SAM areas at 7 days after peptide treatment (*n*≥7). Mock and 1 µM CLV3p were treated as negative and positive controls respectively. Alphabets denote significant differences (two-way ANOVA followed by Tukey’s test, *p*<0.05).

**Figure 3:**
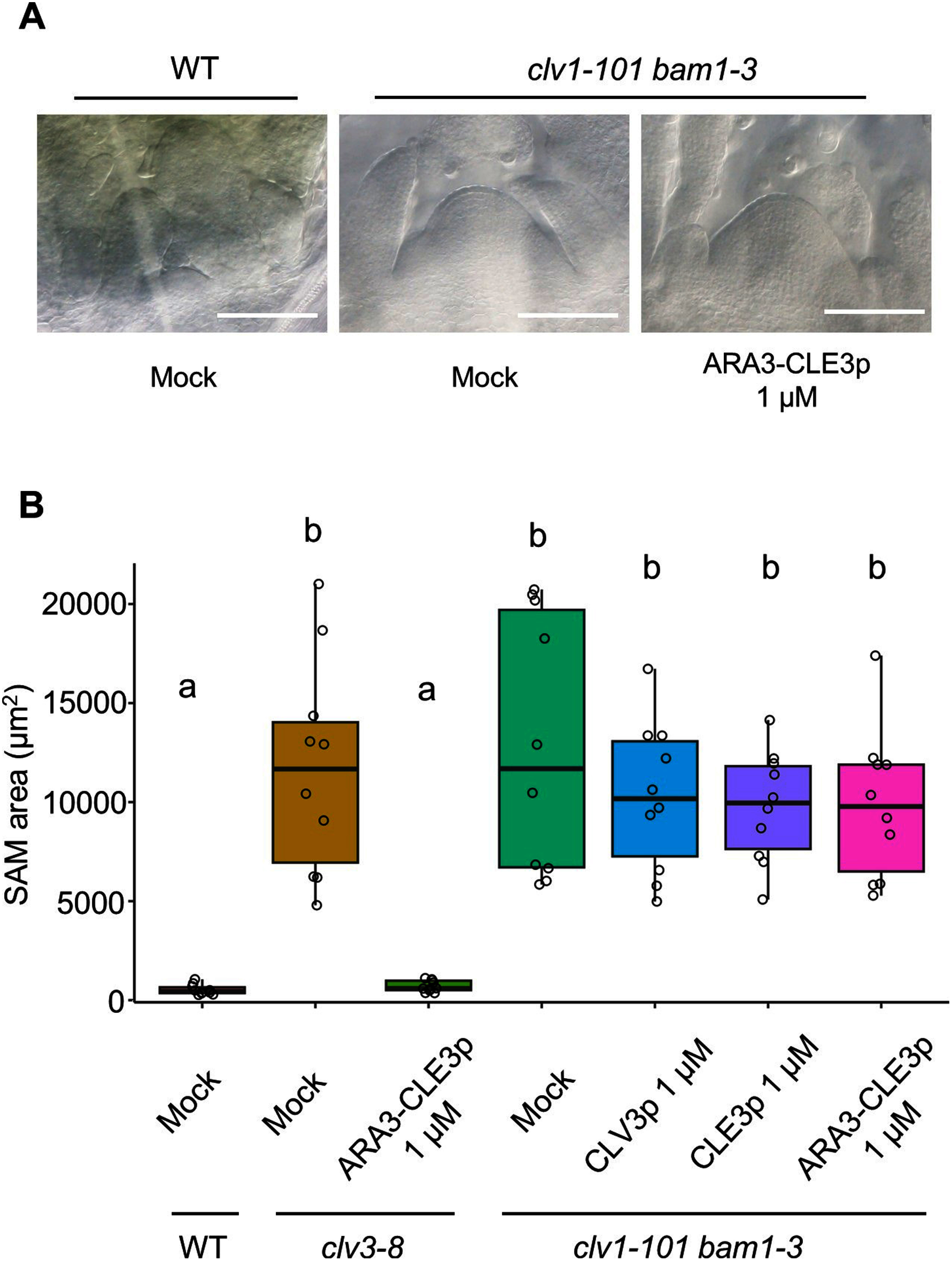
Figure 3. SAM reduction assay in *clv1-101 bam1-3* mutant. (A) Representative micrographs of the SAMs of the wild-type (WT) and *clv1-101 bam1-3* seedlings treated with ARA3-CLE3p. Bar=100 µm. (B) Quantified SAM areas at 7 days after peptide treatment (*n*=10). Mock and 1 µM CLV3p were treated as negative and positive controls respectively. Alphabets denote significant differences (two-way ANOVA followed by Tukey’s test, *p*<0.05).

Our observation highlights the importance of tri-arabinosylation within CLE3 peptide in its bioactivity upon SAM homeostasis. Genetic studies have revealed that genes encoding Hyp O-arabinosyltransferase that catalyzes tri-arabinosylation impact the function of cognate CLE peptides (Kassaw et al. 2017; Ogawa-Ohnishi et al. 2013; Xu et al. 2015; Yoro et al. 2019). Indeed, tri-arabinosylated CLE peptides have been detected in *Arabidopsis* and legume plants (Ohyama et al. 2009; Okamoto et al. 2013, 2015). Moreover, chemically synthesized tri-arabinosylated CLE peptides of *Arabidopsis* and some legume plants have bioactivities cognate receptor-dependently (Corcilius et al. 2017; Hastwell et al. 2019; Imin et al. 2018; Shinohara and Matsubayashi 2013). These lines of evidence suggest that the CLE3 peptide is modified with the tri-arabinose like the other functional peptides. Nevertheless, some CLE peptides have been characterized as non-arabinosylated peptides. For instance, non-arabinosylated form of CLV3 and tri-arabinosylated form are detected from CLV3-overexpressing callus and seedlings respectively, while only non-arabinosylated CLE25 is detected from both culture cells and seedlings (Kondo et al. 2006; Ohyama et al. 2009; Takahashi et al. 2018). Further investigation would reveal the actual mature form of CLE3 peptide. Previous observations that CLE2 and CLE3 peptides delayed lateral root emergence in a CLV1/BAM1-dependent manner suggest that tri-arabinosylation has an impact on lateral root emergence as well (Nakagami et al. 2023a). In this study, we could not exclude the possibility that tri-arabinosylation confers stability on CLE3 peptide as the ARA3-CLE3p was applied for a long-term of 7 days. Further investigation of the short-term effects of ARA3-CLE3p is needed. Increasing our knowledge about CLE signaling will help us better understand plant development, physiological responses, and plant-microbe interactions.

## Description of supplementary files

Materials and Methods.

Supplementary Figure S1. Analytical data for ARA3-CLE3p.

Supplementary Figure S2. SAM reduction assay, related to Figure 2A.
