# Supplementary material for: Tri-arabinosylation facilitates the bioactivity of CLE3 peptide in Arabidopsis
Source: Plant Biotechnol (Tokyo). 2025 Jun 25;42(2):163–6. doi: 10.5511/plantbiotechnology.25.0120b (PMC12235424; doi:10.5511/plantbiotechnology.25.0120b)
Supplement: Supplementary Data [file plantbiotechnology-42-2-25.0120b-s001.pdf]

Supplementary file for

Tri-arabinosylation facilitates the bioactivity of CLE3 peptide in *Arabidopsis*

Satoru Nakagami, Taiki Kajiwara, Hajime Hibino, Taku Yoshiya, Masayoshi Mochizuki, Shugo Tsuda, Toshihiro Yamamoto, Shinichiro Sawa

Corresponding author: Satoru Nakagami, sing_yesterday_for_me1218@yahoo.co.jp

Materials and methods

Plant materials and growth conditions

A. thaliana ecotype Columbia was used as the wild-type plant. The *clv3-8* and *clv1-101 bam1-3* mutants were described previously (Brand et al. 2000; Nakagami et al. 2023). Arabidopsis seeds were stratified for 2 days at 4ºC in the dark, and then incubated for 7 days in the 6-well plate containing B5 liquid medium (pH 5.7) supplemented with 1% (w/v) sucrose and, if needed synthetic peptide, under continuous light (70 µmol s^{-1} m^{-2}) at 23ºC. Ultrapure water was used as a mock treatment. Synthetic peptides were obtained from DGpeptides or PEPTIDE INSTITUTE, Inc.

Peptide bioactivity assay in the SAM

7 days-old seedlings were mounted in chloral hydrate solution (8 g of chloral hydrate, 2 ml of ultrapure water, and 1 ml of glycerol) for 6 hours. Samples were imaged with an Axio Imager M1 microscope (Carl Zeiss) mounted with a DP71 digital camera (Olympus). SAM size was defined as the area above a straight line drawn between the basal edges of the two opposing leaf primordia in a micrograph cross-section. SAM areas were quantified using ImageJ software.

Reference

Brand U, Fletcher JC, Hobe M, et al (2000) Dependence of stem cell fate in Arabidopsis on a feedback loop regulated by CLV3 activity. *Science* 289:617–619. https://doi.org/10.1126/science.289.5479.617

Nakagami S, Aoyama T, Sato Y, et al (2023) CLE3 and its homologs share overlapping functions in the modulation of lateral root formation through CLV1 and BAM1 in Arabidopsis thaliana. *Plant J* 113:1176–1191. https://doi.org/10.1111/tpj.16103

Supplementary Figures

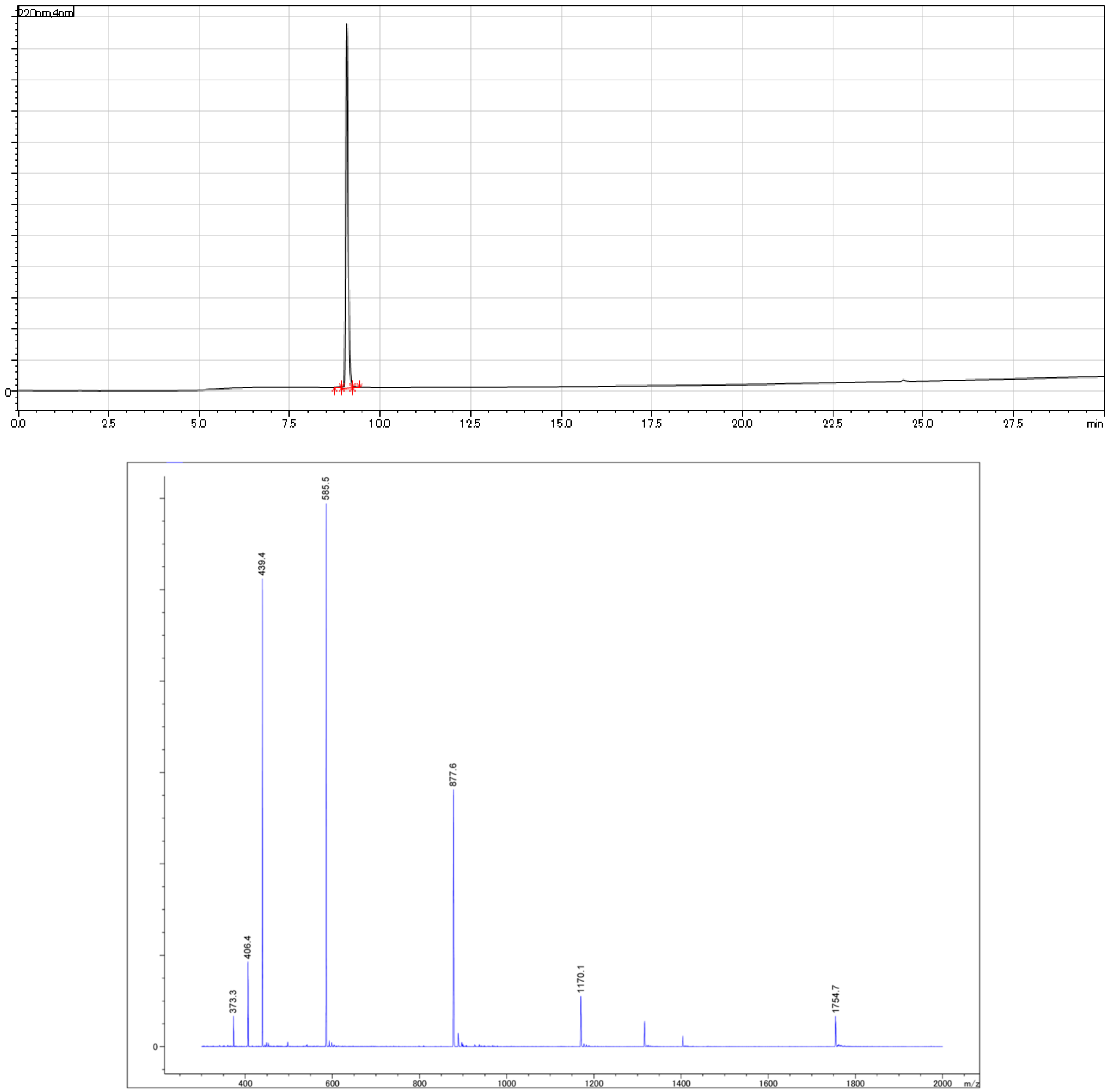

Supplementary Figure S1. Analytical data for ARA3-CLE3p.

Analytical reversed-phase HPLC was performed using a Shimazu Nexera Lite with detection at 220 nm. The analysis was conducted using a YMC-ODS AA12S03-1546WT column at a flow rate of 1 mL/min [mobile phase: 1–60% MeCN (0.1% TFA) in H_2O (0.1% TFA) over 25 min]. Low-resolution ESI+ mass spectra (LRMS) were measured using an Agilent 1260 Infinity II and LC/MSD XT (G6135B) system. Analytical HPLC: RT 9.1 min (1 to 60% MeCN [0.1% TFA] in H_2O [0.1% TFA] over 25 min, sample dissolved in H_2O, λ = 220 nm). MS: m/z 878 $[M+2H]^{2+}$, 586 $[M+3H]^{3+}$, 439 $[M+4H]^{4+}$.

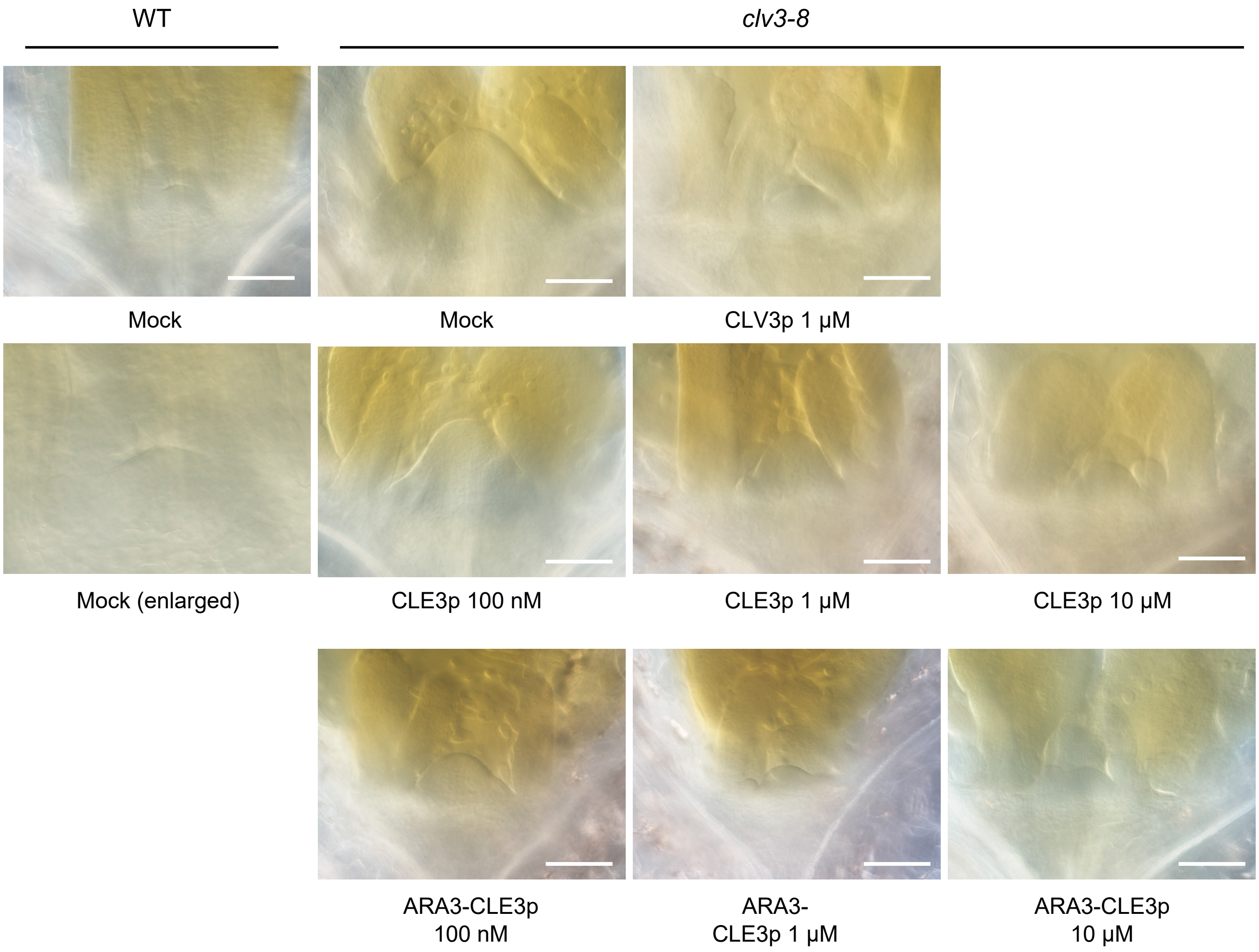

Supplementary Figure S2. SAM reduction assay, related to Figure 2A.

Representative micrographs of the SAMs of the wild-type (WT) and *clv3-8* seedlings treated with synthetic peptides. Bar = 100 μm. Related to Figure 2A.
